# Psychological Impact of the COVID-19 Pandemic on Healthcare Professionals in Tunisia: Risk and Protective Factors

**DOI:** 10.3389/fpsyg.2021.754047

**Published:** 2021-12-14

**Authors:** Ahmed Sami Hammami, Mohamed Jellazi, Lobna Mahjoub, Maya Fedhila, Sonia Ouali

**Affiliations:** ^1^Department of Internal Medicine, CHU F Bourguiba Monastir, Monastir, Tunisia; ^2^Biochemistry Laboratory, LR12ES05 LR-NAFS “Nutrition – Functional Food and Vascular Health”, Faculty of Medicine, University of Monastir, Monastir, Tunisia; ^3^Faculty of Dental Medicine of Monastir, Monastir, Tunisia

**Keywords:** psychological symptoms, mental health, healthcare professionals, COVID-19 pandemic, risk factors, protective factors

## Abstract

**Background:** Our study aimed to evaluate the magnitude of different psychological outcomes among Tunisian healthcare professionals (HCPs) during the first wave of the coronavirus disease 2019 (COVID-19) pandemic, and to identify the associated factors.

**Methods:** Healthcare professionals completed a cross-sectional questionnaire during a 3-week period in the first wave of the COVID-19 pandemic in Tunisia. The survey collected demographic information, factors that may interfere with the psychological outcomes, behavioral changes, and mental health measures. Mental health was assessed using three scales: the Seven-Item Insomnia Severity Index, the Two-Item Patient Health Questionnaire, and the Two-Item Generalized Anxiety Disorder instrument. Multivariable logistic regression was conducted to identify factors associated with psychological outcomes.

**Results:** A total of 503 HCPs successfully completed the survey, and 493 agreed to enroll in the study: 411 (83.4%) physicians, 323 (64.2%) women, and 271 (55%) with a second-line work position. A significant proportion of HCPs had anxiety (35.7%), depression (35.1%), and insomnia (23.7%). Women, those with a psychiatric history, and those using public transportation had higher proportions for overall symptoms compared with other groups, for example, depression in 44.9% of female participants vs. 18.2% of male participants (*p* = 0.00). Those with a previous medical history and nurses had more anxiety and insomnia compared with other groups, for example, anxiety in 45.1% of nurses, 36.1% of interns/residents, and 27.5% of attending physicians (*p* = 0.04). Multivariable logistic regression showed that female gender was a risk factor for all psychological outcomes, whereas psychiatric history was a risk factor for both anxiety and insomnia [odds ratio (OR) = 2.86, 95% CI 1.78–4.60, *p* = 0.00 for insomnia]. Using protective equipment was associated with a lower risk for depression (OR = 0.41, 95% CI 0.27–0.62, *p* = 0.00) and anxiety. Physical activity was also protective against depression and anxiety (OR = 0.41, 95% CI 0.25–0.67, *p* = 0.00).

**Conclusion:** Psychological symptoms are usually overlooked or dismissed by HCPs, although the COVID-19 pandemic played a major role in exacerbating this burden. Prompt psychological support should be endorsed and simple measures, such as physical activity and ensuring the availability of personal protective equipment, are paramount to improve mental health outcomes and the quality of care provided to patients.

## Introduction

At the end of December 2019, a novel coronavirus was reported to be the cause of a myriad of pneumonia cases in the Chinese city of Wuhan. The responsible agent was a virus called severe acute respiratory syndrome coronavirus 2 (SARS-CoV-2). In this article, we will refer to the associated disease as the coronavirus disease 2019 (COVID-19) (Lai et al., 2020).

This rapidly spreading infection resulted in an epidemic throughout the entire region and subsequently the entire country of China, followed by a surging number of cases in other countries around the world, which progressively overwhelmed healthcare systems worldwide (Armocida et al., 2020; Xie et al., 2020). By the beginning of March 2020, it was identified as a pandemic (WHO, 2019). Consequently, the WHO highlighted the excessively high burden on healthcare professionals (HCPs), and called for an intervention to address the immediate needs and prevent serious impacts on both physical and mental health in this particular population (Covid 19 Public Health Emergency of International Concern (PHEIC), 2020).

Tunisia was not an exception to the spread of the pandemic, with the first case recorded on March 02, 2020 (Corona virus cases in Tunisia, 2020). As in other affected countries, psychological distress began to propagate, and quickly expanded among HCPs who are directly or indirectly involved in the diagnosis, treatment, and care of patients with COVID-19, and are therefore at high risk of infection (Xiao et al., 2020). Tunisia faced a specific challenge in preparing for the emerging pandemic: a context of political and economic fragility with a precarious financial situation following the democratic transition resulted in the deterioration of the healthcare system and decreasing motivation among HCPs (Zedini et al., 2016; Weilandt, 2018). Among the factors reported to contribute to the mental health burden of these healthcare workers are the overwhelming spread of the life-threatening disease, the gradual increase in numbers of newly diagnosed and suspected cases, the surge in mortality rates, the fear of becoming infected and transmitting the infection to loved ones, the enormous workload, the potential lack of personal protective equipment (PPE), the depletion of certain drugs, the lack of an effective treatment, and feelings of being inadequately supported (Muller et al., 2020). Accordingly, it has been reported that during previous viral outbreaks, HCPs endured a high degree of both physical (Xiao et al., 2020) and mental stress during and even years after the end of epidemics (Lancee et al., 2008). The current pandemic has had several psychiatric consequences among healthcare practitioners who treat patients with confirmed or suspected COVID-19 infection (Xiang et al., 2020). Clearly, HCPs should be regarded as a high-risk population because of their higher anxiety, depression, and insomnia levels compared with the general population (Vindegaard and Benros, 2020). In addition, occupational stress in HCPs may exacerbate previously diagnosed psychological problems, and can lead to long-term psychological consequences; most importantly, it may adversely affect the quality of healthcare provided to patients.

A large number of studies in many different countries describe the prevalence of mental health sequelae associated with the COVID-19 pandemic (Brailovskaia et al., 2021). Those research have documented the magnitude of the psychological burden among healthcare workers (Wei et al., 2020; Busch et al., 2021), and have identified risk and protective factors (Wei et al., 2020; Brailovskaia et al., 2021). For instance, an Italian cross-sectional survey-based study with a retrospective assessment showed that the COVID-19 outbreak led to life changes in Italian healthcare workers in terms of increase in negative mood, worry, loneliness, fatigue, restlessness, and decrease in happiness (Mansueto et al., 2021). However, none of those studies provided data about the impact of this pandemic on mental health in the Maghreb region. In Tunisia, maintaining health workers’ motivation is particularly challenging against a background of low levels of trust in the health system and the government, and this is also the case in other areas of the Middle East and North Africa (MENA) region where governments are perceived to be corrupt. Therefore, estimating the prevalence of risk factors in this population is important to guide public health measures intended to protect health professionals and their families, and to maintain a functioning healthcare system.

Addressing the needs of frontline HCPs during the COVID-19 pandemic is an important priority (Remuzzi and Remuzzi, 2020). Accordingly, the aims of our study were to screen for depression, anxiety, and insomnia among Tunisian HCPs during the first wave, and to detect potential risk factors associated with these psychological outcomes. Our findings will, we hope, help to maintain a sustainable, robust healthcare response to the pandemic while safeguarding the wellbeing of HCPs in our country and elsewhere.

## Materials and Methods

We conducted an online cross-sectional survey at a crucial time when the first wave of the COVID-19 pandemic was sweeping North Africa and Tunisia.

The eligibility criteria were employment as an HCP at any type of public or private health institution in all 24 Tunisian governorates that were registered with the Ministry of Health, and consent for data collection and analysis.

To reach a large number of HCPs, we released information about our survey through the official mailing list of the medical and paramedical professional network in Tunisia, and through health professional groups on Facebook. This approach allowed us to reach large numbers of survey respondents quickly and continuously in all governorates.

All participants were required to provide their consent electronically before responding to the questionnaire. Those who agreed were directed to the questionnaire, and those who declined automatically exited the survey.

Participants were required to respond to all the questions to submit their survey.

All 24 Tunisian governorates were involved, and the responses were then reassembled into three main subgroups according to the Tunisian health department, depending on the prevalence rate in each governorate, i.e., low-, moderate-, or high-prevalence regions (Corona virus cases in Tunisia, 2020).

This study received approval from the research ethics committee of the Faculty of Medicine of Monastir, Tunisia, before the survey was conducted.

The questionnaire was an Internet-based survey containing four sections: basic demographic information, factors that may interfere with psychological outcomes, psychological impact questions, and psychological screening tests.

### Demographics

Data were collected for age (years), gender (male or female), marital status (married or unmarried), medical and psychological history, type of hospital (primary or secondary care center, or tertiary care hospital), technical title (intern/resident, attending/primary care physician, or nurse), and prevalence at the work location (low, moderate, or high).

### Factors That May Interfere With Psychological Outcomes

These factors include work position (frontline for those who work on wards with COVID-19-positive patients and those who staff fever clinic nightshifts, second line for those working in other departments), working in the fever clinic (yes or no), type of transportation used to travel to work (public or private), having children (yes or no), living with family (yes or no), physical activity (yes or no), and the availability of PPE (yes or no).

Fever clinics are facilities usually established outside an emergency department to perform triage and classify patients according to their likelihood of having COVID-19 infection. These clinics have the authority to hospitalize high-risk patients as an interim solution until their subsequent assignment to a specialized unit.

Physical activity in the form of moderate aerobic exercise or more vigorous activity was considered activity performed two or more days per week and lasting at least 40 min per session. The type of physical activity was not specified in this survey.

### Psychological Impact Questions

These items inquired about experiencing nightmares related to the virus, and about noticeable changes in how respondents performed regular physical examinations for patients.

### Psychological Screening Tests

We used three tests to screen for potential mental disorders among HCPs: the Seven-Item Insomnia Severity Index (ISI), the Two-Item Patient Health Questionnaire (PHQ-2), and the Two-Item Generalized Anxiety Disorder instrument (GAD-2).

The ISI is a concise self-reporting tool that measures the respondent’s perception of insomnia. Scores range from 0 to 28, with 0–7 indicating no significant insomnia, 8–14 indicating subthreshold insomnia, 15–21 indicating moderate insomnia, and 22–28 indicating severe insomnia (Morin et al., 2011).

We defined clinically significant insomnia as a score ≥ 15 (merging the latter two categories), and non-clinically significant insomnia as a score < 15 (merging the first two categories).

Depression and anxiety were assessed with the PHQ-2 and the GAD-2 instrument, respectively. A score of three points was the preferred cut-off to positively screen for depression (Löwe et al., 2010) and anxiety (Kroenke et al., 2007). We divided participants into 2 groups: those who screened positively for depression and anxiety (score ≥ 3) and those whose score was < 3.

### Statistical Analysis

All data were analyzed with SPSS statistical software, version 26.0 (IBM Corp, Monastir, Tunisia). The significance level was set at *p* ≤ 0.05, and all tests were two-tailed. The ranked data were calculated from the counts of each level of symptoms of anxiety, depression, and insomnia in accordance with the pre-established scales we used, and all descriptive data are expressed as numbers (*n*) and frequencies (%). Quantitative variables were compared between two groups with the *t*-test, and between more than two groups with analysis of variance. Categorical variables were compared with Fisher’s exact test. Univariate analysis was used to evaluate candidate predictors of mental health disorders, and variables with *p* values < 0.2 were selected for the multivariable analysis. The associations between outcomes and risk factors are presented as odds ratios (OR) and 95% CI, after adjustment for confounders.

## Results

The initial total number of participants was 503 (100%), of whom 10 (2%) declined to participate in the survey. The final number of participants was 493.

### Demographic Characteristics

The characteristics of the participants are presented in Table 1.

**TABLE 1:** Demographic and occupational characteristics of participants.

	No. (%)								

**Characteristics**		**Workplace (COVID risk)**			**Occupation**		**Technical title (3 subgroups)**		
	**Total**	**Low**	**Moderate**	**High**	**Physician**	**Nurse**	**Intern/Resident**	**Attending**	**Nurse**
*Overall*	493 (100)	96 (19.5)	234 (47.5)	163 (33.1)	411 (83.4)	82 (16.6)	302 (61.3)	109 (22.1)	82 (16.6)
*Gender*									
Male	170 (33.8)	38 (39.6)	74 (31.6)	58 (35.6)	145 (35.3)	25 (30.5)	97 (32.1)	48 (44)	25 (30.5)
Female	323 (64.2)	58 (60.4)	160 (68.4)	105 (64.4)	266 (64.7)	57 (69.5)	205 (67.9)	61 (56)	57 (69.5)
*Age (years)*									
<30	330 (66.9)	58 (60.4)	165 (70.5)	107 (65.6)	284 (69.1)	46 (56.1)	265 (87.7)	19 (17.4)	46 (56.1)
≥30	163 (33.1)	38 (39.6)	69 (29.5)	56 (34.4)	127 (30.9)	36 (43.9)	37 (12.3)	90 (82.6)	36 (43.9)
*Marital status*									
Unmarried	351 (69.8)	65 (67.7)	168 (71.8)	118 (72.4)	299 (72.7)	52 (63.4)	252 (83.4)	47 (43.1)	52 (63.4)
Married	142 (28.2)	31 (32.3)	66 (28.2)	45 (27.6)	112 (27.3)	30 (36.6)	50 (16.6)	62 (56.9)	30 (36.6)
Pregnant	5 (1)	2 (2.1)	1 (0.4)	2 (1.2)	5 (1.2)	0 (0)	3 (1)	2 (1.8)	0 (0)
*Children*									
Yes	113 (22.5)	25 (26)	52 (22.2)	26 (22.1)	86 (20.9)	27 (32.9)	24 (7.9)	62 (56.9)	27 (32.9)
No	380 (75.5)	71 (74)	182 (77.8)	127 (77.9)	325 (79.1)	55 (67.1)	278 (92.1)	47 (43.1)	55 (67.1)
*Living with family*									
No	202 (41)	37 (38.5)	95 (40.6)	70 (42.9)	186 (45.3)	16 (19.5)	160 (53)	26 (23.9)	16 (19.5)
Yes	291 (59)	59 (61.5)	139 (59.4)	93 (57.1)	225 (54.7)	66 (80.5)	142 (47)	83 (76.1)	66 (80.5)
*Medical history*									
Yes	106 (21.5)	22 (22.9)	49 (20.9)	35 (21.5)	94 (22.9)	12 (14.6)	58 (19.2)	36 (33)	12 (14.6)
No	387 (78.5)	74 (77.1)	185 (79.1)	128 (78.5)	317 (77.1)	70 (85.4)	244 (80.8)	73 (67)	70 (85.4)
*Psychiatric history*									
Yes	63 (12.8)	12 (12.5)	34 (14.5)	17 (10.4)	54 (13.1)	9 (11)	45 (14.9)	9 (8.3)	9 (11)
No	430 (87.2)	84 (87.5)	200 (85.5)	146 (89.6)	357 (86.9)	73 (89)	257 (85.1)	100 (91.7)	73 (89)
*Work position*									
Frontline	222 (45)	47 (49)	111 (47.4)	64 (39.3)	176 (42.8)	46 (56.1)	140 (46.4)	36 (33)	46 (56.1)
Second line	271 (55)	49 (51)	123 (52.6)	99 (60.7)	235 (57.2)	36 (43.9)	162 (53.6)	73 (67)	36 (43.9)
*Type of hospital*									
Primary and secondary care center	130 (26.4)	31 (32.3)	62 (26.5)	37 (22.7)	90 (21.9)	40 (48.8)	23 (7.6)	67 (61.5)	40 (48.8)
Tertiary care center	363 (73.6)	65 (67.7)	47.4 (73.5)	126 (77.3)	321 (78.1)	42 (51.2)	279 (92.4)	42 (38.5)	42 (51.2)

The final number of participants enrolled in this study was 493, of whom 411 (83.4%) were physicians and 82 (16.6%) were nurses. The majority of respondents (47.5%) worked in moderate-prevalence regions. Most participants were women (323, 64.2%), were younger than 30 years old (330, 66.9%), were unmarried (351, 69.8%), and worked in tertiary care centers (363, 73.6%). In most participants, there was no history of medical (387, 78.5%) or psychiatric illness (430, 87.2%), and a majority worked as second-line HCPs (271, 55%).

### Factors Associated With Psychological Findings

A significant proportion of HCPs had depression (35.1%), anxiety (35.7%), and insomnia (23.7%); overall, at least one of these psychological outcomes was present in 45.8% of HCPs. Women, respondents who had a history of psychiatric illness, and HCPs who used public transportation had higher overall frequencies for all symptoms. Anxiety was noted by 44.9% of women vs. 18.2% of men, and insomnia was noted by 29.1% of women vs. 13.5% of men (both *p* = 0.00). Among participants with a history of psychiatric illness, anxiety was noted by 63.5% vs. 31.6% of those with no such history (*p* = 0.00), and depression was noted by 54.0% of the former vs. 32.3% of the latter (*p* = 0.01). Among those who used public transportation, anxiety was present in 50.4% vs. 31.2% of participants who used private transportation (*p* = 0.00) (Table 2). Among HCPs who were ≥ 30 years old, symptoms of anxiety were more frequent (73.0%) compared with younger participants (60.0%, *p* = 0.00).

**TABLE 2:** Anxiety, depression, and insomnia measures in the total cohort and subgroups.

Variables	PHQ-2, depression			GAD-2, anxiety			ISI, insomnia		
	Depressed	Not depressed	*p* value	Anxiety	No anxiety	*p* value	Insomnia	No insomnia	*p* value
*Total*	173 (35.1)	320 (64.9)		176 (35.7)	317 (64.3)		117 (23.7)	367 (76.3)	
*Age (years)*									
<30	122 (37)	208 (63)	0.214	198 (60)	132 (40)	<0.01	74 (22.4)	256 (77.6)	0.331
≥30	51 (31.3)	112 (68.7)		119 (73)	44 (27)		43 (26.4)	120 (73.6)	
*Gender*									
Male	42 (24.7)	128 (75.3)	<0.01	31 (18.2)	139 (81.8)	<0.01	23 (13.5)	147 (86.5)	<0.01
Female	131 (40.6)	192 (59.4)		145 (44.9)	178 (55.1)		94 (29.1)	229 (70.9)	
*Medical history*									
Yes	44 (41.5)	62 (58.5)	0.118	48 (45.3)	58 (54.7)	0.02	34 (32.1)	72 (67.9)	0.023
No	129 (33.3)	258 (66.7)		128 (33.1)	259 (66.9)		83 (21.4)	304 (78.6)	
*Psychiatric history*									
Yes	34 (54)	29 (46)	<0.01	40 (63.5)	23 (36.5)	<0.01	33 (52.4)	30 (47.6)	<0.01
No	139 (32.3)	291 (67.7)		136 (31.6)	294 (68.4)		84 (19.5)	346 (80.5)	
*Work position*									
Frontline	88 (39.6)	134 (60.4)	0.055	76 (34.2)	146 (65.8)	0.539	62 (27.9)	160 (72.1)	0.047
Second line	85 (31.4)	186 (68.6)		100 (36.9)	171 (63.1)		55 (20.3)	216 (79.7)	
*Fever clinic*									
Yes	65 (41.1)	93 (58.9)	0.034	97 (61.4)	61 (38.6)	0.204	46 (29.1)	112 (70.9)	0.036
No	108 (32.2)	227 (67.8)		220 (65.7)	115 (34.3)		71 (21.2)	264 (78.8)	
*Technical title*									
Interns and residents	103 (34.1)	199 (65.9)	0.161	109 (36.1)	193 (63.9)	0.042	60 (19.9)	242 (80.1)	<0.01
Attending and primary care physicians	34 (31.2)	75 (68.8)		30 (27.5)	79 (72.5)		23 (21.1)	86 (78.9)	
Nurses	36 (43.9)	46 (56.1)		37 (45.1)	45 (54.9)		34 (23.7)	48 (76.3)	
*Protective equipment*									
Yes	71 (26.4)	198 (73.6)	<0.01	80 (29.7)	189 (70.3)	<0.01	53 (19.7)	216 (80.3)	0.021
No	102 (45.5)	122 (54.5)		96 (42.9)	128 (57.1)		64 (28.6)	160 (71.4)	
*Physical activity*									
Yes	39 (24.8)	118 (75.2)	<0.01	36 (22.9)	121 (77.1)	<0.01	28 (17.8)	129 (82.2)	0.035
No	134 (39.9)	202 (60.1)		140 (41.7)	196 (58.3)		89 (26.5)	247 (73.5)	
*Children*									
Yes	36 (31.9)	77 (68.1)	0.434	30 (26.5)	83 (74.3)	0.021	29 (25.7)	84 (74.3)	0.583
No	137 (36.1)	243 (63.9)		146 (38.4)	234 (61.6)		88 (23.2)	292 (76.8)	
*Public transportation*									
Yes	54 (47)	61 (53)	<0.01	58 (50.4)	57 (49.6)	<0.01	35 (30.4)	80 (69.6)	0.037
No	119 (31.5)	259 (68.5)		118 (31.2)	260 (68.8)		82 (21.7)	296 (78.3)	
*Living with family*									
Yes	102 (59)	71 (41)	0.529	113 (38.83)	178 (61.17)	0.049	73 (25.1)	218 (74.9)	0.23
No	189 (59.1)	131 (40.9)		63 (31.18)	139 (68.81)		44 (21.8)	158 (78.2)	

A previous history of medical illness and being employed as a nurse were both associated with a higher frequency of anxiety and insomnia compared with employment as an intern or resident, or as an attending physician. Anxiety was found in 45.1% of nurses vs. 36.1% of interns/residents and 27.5% of attending physicians (*p* = 0.04), and insomnia was seen in 23.7% of nurses, 19.9% of interns/residents, and 21.1% of attending physicians (*p* = 0.00). In addition, participants with a history of medical illness showed more anxiety and insomnia compared with those with no such history. Among the former, anxiety was seen in 45.3% vs. 33.1% of the latter (*p* = 0.02). Insomnia was seen in 32.1% of respondents with a previous medical history vs. 21.4% of those with no such history (*p* = 0.02).

Healthcare professionals employed in frontline positions had more frequent insomnia (27.9%) than those working in second-line positions (20.3%, *p* = 0.04). Individuals working in fever clinics were more prone to have symptoms of depression (41.1% vs. 32.2%, *p* = 0.03) and insomnia (29.1% vs. 21.2%, *p* = 0.03) compared with those who did not work in this clinical setting. We highlight the finding that 33.7% of all participants had nightmares related to the virus, and 66.3% reported behavioral changes in performing routine clinical examinations of their patients.

Compared with those who lived alone, HCPs who lived with their family were more prone to anxiety. Among HCPs living with their family, anxiety was found in 38.8% vs. 31.18% of those who lived alone (*p* = 0.04).

Physical activity and the availability of PPE were associated with a lower frequency of psychiatric outcomes. Anxiety was noted in 22.9% of participants who were physically active vs. 41.7% of those who were not (*p* = 0.00). Depression was noted in 26.4% of those who had access to PPE vs. 45.5% of those who did not (*p* = 0.00).

Multivariable logistic regression (Table 3), after confounders were controlled, identified the following risk factors for depression: female gender (OR 1.88, 95% CI 1.21–2.92, *p* = 0.01) and using public transportation (OR 1.69, 95% CI 1.06–2.69, *p* = 0.03). As protective factors, this analysis identified physical activity (OR 0.49, 95% CI 0.31–0.78, *p* = 0.00) and availability of appropriate PPE (OR 0.41, 95% CI 0.27–0.62, *p* = 0.00).

**TABLE 3:** Risk and protective factors for mental health outcomes identified by multivariable regression.

Variable	No. cases/No. total cases (%)	Adjusted OR (95% CI)	*p* value
**PHQ-2, depression symptoms**			
*Gender*			
Men	42/170 (24.7)	1 (reference)	0.005
Women	131/323 (40.6)	1.88 (1.21–2.92)	
*Physical activity*			
Yes	39/157 (24.8)	0.49 (0.31–0.78)	0.002
No	134/336 (39.9)	1 (reference)	
*Protective equipment*			
Yes	71/269 (26.4)	0.41 (0.27–0.62)	0.000
No	102/124 (45.5)	1 (reference)	
*Public transportation*			
Yes	54/115 (47)	1.69 (1.06–2.69)	0.027
No	119/378 (31.5)	1 (reference)	
**GAD-2, Anxiety symptoms**			
*Gender*			
Men	31/170 (18.2)	1 (reference)	0.000
Women	145/323 (44.9)	2.86 (1.78–4.60)	
*Psychiatric history*			
Yes	40/63 (63.5)	3.05 (1.66–5.63)	0.000
No	136/430 (31.6)	1 (reference)	
*Physical activity*			
Yes	36/157 (22.9)	0.41 (0.25–0.67)	0.000
No	140/336 (41.7)	1 (reference)	
*Protective equipment*			
Yes	80/269 (29.7)	0.65 (0.43–0.98)	0.044
No	96/224 (42.9)	1 (reference)	
*Type of hospital*			
Primary and secondary care center	53/130 (40.8)	1.76 (1.01–3.08)	0.045
Tertiary care center	123/363 (33.9)	1 (reference)	
*Living with family*			
Yes	113/291 (64.2)	1.62 (1.02–2.60)	0.041
No	63/202 (35.8)	1 (reference)	
*Public transportation*			
Yes	58/115 (50.4)	1.73 (1.06–2.83)	0.029
No	118/378 (31.2)	1 (reference)	
**ISI, insomnia symptoms**			
*Gender*			
Men	23/170 (13.5)	1 (reference)	0.002
Women	94/323 (29.1)	2.29 (1.34–3.92)	
*Psychiatric history*			
Yes	33/63 (52.4)	3.82 (2.09–6.96)	0.000
No	84/430 (19.5)	1 (reference)	

Five risk factors were independently related to anxiety among HCPs: history of psychiatric illness (OR 3.05, 95% CI 1.66–5.63, *p* = 0.00), female gender (OR 2.86, 95% CI 1.78–4.60, *p* = 0.00), working in primary or secondary care centers (OR 1.76, 95% CI 1.01–3.08, *p* = 0.05), using public transportation (OR 1.73, 95% CI 1.06–2.83, *p* = 0.03), and living with family (OR 1.62, 95% CI 1.02–2.60, *p* = 0.04). Conversely, the factors found to be protective against anxiety were physical activity (OR 0.41, 95% CI 0.25–0.67, *p* = 0.00) and PPE (OR 0.65, 95% CI 0.43–0.98, *p* = 0.04).

Regarding insomnia, we identified two risk factors. A history of a psychiatric illness showed the strongest association (OR 3.82, 95% CI 2.09–6.96, *p* = 0.00), followed by female gender (OR 2.29; 95% CI 1.34–3.92, *p* = 0.00).

## Discussion

This study examined the presence of anxiety, depression, and insomnia symptoms among health workers in Tunisia during the early phase of the COVID-19 pandemic. During the previous decade, Tunisia faced few epidemics without a significant impact on its healthcare system. The 2012 West Nile virus epidemics (Hammami et al., 2017) and the 2013 MERS-COV outbreaks (Abroug et al., 2014) are two examples, but neither of them was comparable to the COVID-19 pandemic in terms of amplitude and consequences on country-wide aspects of healthcare, economy, the agri-food system, and households (Zouhair et al., 2020).

The current pandemic emerged in Tunisia at a time when the country was grappling with an economic crisis, in addition to the political instability (Weilandt, 2018). In fact, Tunisia is facing an unprecedented crisis due to continuous political turmoil and the unfolding economic and financial meltdown, exacerbated by the COVID-19 pandemic.

The pandemic started with a significant delay compared with the European countries that had already reached the exponential phase of infection distribution. However, in the setting of a fragile healthcare system, Tunisian HCPs found themselves unprepared either psychologically or logistically to face this rapidly evolving pandemic (Chaari and Golubnitschaja, 2020), especially at its first onset.

Furthermore, as a lower middle-income country (Classification of countries according to their income, 2019), Tunisia has very limited resources; hence, HCPs struggled with a lack of available tests and with discrepancies in test performance between different regions, which led to a heterogeneous picture of the infection distribution and mortality rates (Chaari and Golubnitschaja, 2020). They also faced difficulties due to insufficient medical equipment and PPE, and limited hospitalization capacity (Fredj and Chérif, 2020). These factors consequently made HCPs susceptible to negative mental health outcomes.

This study is the first of its kind in the Maghreb region. Countries in this region (Algeria, Morocco, Tunisia, and Libya) are characterized by generally similar healthcare systems, thus our sample may reflect, to some extent, the mental health situation among HCPs during this pandemic in other Maghreb countries.

This Tunisian study characterizes the impact of the pandemic during its early phase on mental health in HPCs, including a wide range of medical professionals from all over the country. Previous studies in different parts of the world were cross-sectional or longitudinal, and included essentially doctors and nurses. A meta-analysis of this earlier research showed that on average, two out of every five HCPs endured negative psychological outcomes during the COVID-19 pandemic, for example, anxiety, depression, insomnia, and other worrisome outcomes (Muller et al., 2020). Another meta-analysis was undertaken on a global scale combined studies of influenza and coronavirus epidemics, and reported substantial proportions of psychological symptoms among HCPs (Serrano-Ripoll et al., 2020). Thus, HCPs, worldwide have been challenged psychologically in the context of the current global health crisis.

As previously highlighted in other research (Lai et al., 2020), our survey documented significant frequencies of psychological symptoms among HCPs during the COVID-19 pandemic: 35.7% had anxiety, 35.1% had depression, and 23.7% had insomnia. Fekih-Romdhane et al. (2020) conducted a cross-sectional study among solely residents (*n* = 210) in Tunisia, and the Depression, Anxiety and Stress Scale (DASS) scores they obtained disclosed severe or extremely severe levels of depression in 30.5%, anxiety in 24.3%, and stress in 18.6% of their participants. The ISI scores in their study revealed a high prevalence rate of insomnia (41.4%) (Fekih-Romdhane et al., 2020). Similarly, Dong and Gao (2021) reported a high prevalence of anxiety (24.15%) and insomnia (39.83%). These authors used the Self-Reported Anxiety Scale (SAS) and the ISI to screen for anxiety and insomnia, respectively.

In a study by Khanal et al. (2020), health workers in Nepal completed an online questionnaire; the findings disclosed symptoms of anxiety in 41.9%, depression symptoms in 37.5%, and symptoms of insomnia in 33.9%. Anxiety and depression were measured with the 14-item Hospital Anxiety and Depression Scale (HADS: 0–21), and insomnia was measured with the seven-item ISI (range 0–28).

Pappa et al. (2020) in their systematic review and meta-analysis of 13 cross-sectional studies (*n* = 33,062), reported a high prevalence rate of anxiety, depression, and insomnia during the COVID-19 pandemic. On the other hand, other researchers reported much lower rates. In Singapore, only 14.5% of HCPs screened positive for anxiety, and only 8.9% had depression. These results may be explained by the fact that new protective equipment was available in Singapore to protect HCPs and help them deal with the COVID-19 calamity (Tan et al., 2020). Differences in prevalence rates between studies can also be explained by the different settings where each study was conducted. Another contributing factor may be the use of different scoring scales.

In the present study, health workers who were women, and those with a history of mental health problems, had a higher odds of exhibiting anxiety, depression, and insomnia symptoms compared with men and to participants with no such history. Similar findings were observed in other studies (Lai et al., 2020; Prasad et al., 2021). The difference between genders may be explained in part by women’s greater propensity to express psychological distress compared with men (Zhang and Wing, 2006; McLean et al., 2011; Abate, 2013; Mansueto and Faravelli, 2021). These findings may also reflect the predominance of women in patient-facing roles. Other factors that can affect women healthcare workers’ wellbeing include role strain, difficulties maintaining work–life balance, the consequences associated with family members, gender-related discrimination, and a lack of sufficient support systems.

Healthcare professionals with a history of mental illness experienced more anxiety and insomnia, which is consistent with the results of Zhang W.R. et al. (2020). Khanal et al. (2020) conducted a cross-sectional study among 475 health workers who treated patients with COVID-19 in Nepal and noted that a history of medication for mental health problems was significantly associated with a higher likelihood of experiencing symptoms of anxiety, depression, and insomnia compared with colleagues with no such history.

A similar finding was observed in a study by Zhu et al. (2020) who noted that workers with a history of mental health problems were more likely to have anxiety, depression, and stress. A recent study of 1,685 participants in the USA showed that HCPs with a history of psychiatric illness were at a greater risk for significant mental burden (Young et al., 2021). In fact, HCPs with a mental illness are inherently predisposed and more vulnerable to the appearance of psychiatric symptoms because of their mental fragility, and hence their lower threshold for distress.

Healthcare professionals with medical history experienced more anxiety and insomnia which is consistent with the results of Zhang W.R. et al. (2020). We believe that this is due to their assumption of having potentially worse outcomes if they get infected with COVID-19 (Wei et al., 2020; Wolff et al., 2020), especially, during the first wave when there was no previously established information about the COVID-19 sequelae and its interference with other illnesses. Our findings are supported by Zhang et al., who showed that having an organic disease was an independent factor for insomnia, anxiety, and depression.

Based on the multivariable regression model, we found that female gender and psychiatric history were independent risk factors for mental health outcomes in nearly all measures.

In the present study, nurses reported more symptoms of anxiety and insomnia than their colleagues (69.5% of whom were women). Nurses are considered essential HCPs deeply involved in patient management with close, constant physical proximity (e.g., repeated monitoring and recording signs, drug administration, and blood sampling, etc.) and hence frequently exposed to the highest risk of contagion (Barrett et al., 2020). Similarly, another highly exposed group were those working as frontline HCPs or in fever clinics, who experienced more insomnia than second-line workers. These subgroups are responsible for providing direct care to patients with COVID-19 and for collecting sputum specimens for virus detection.

Interestingly, we found that using public transportation to go to work was a source of worse mental health outcomes in all measures, and it was shown by multivariable regression that public transport was a major risk factor for depression and anxiety. These results are mainly due to the heightened stress level caused by the risk of contagion when in close contact with people who might be infected (Arora et al., 2020).

Similarly, HCPs who were in close contact with their family members expressed heightened levels of anxiety compared with those who did not live with their families. This finding is attributable to the fear of being COVID-19-positive and the consequent risk of transmitting the virus to their families.

Coping psychologically with a pandemic can be arduous for healthcare workers (Wong et al., 2005). The origin of psychological issues may be related with fear of infection, fear of transmitting the virus to their relatives (we note that residing with family members was an independent risk factor for anxiety in our participants), lack of information about the virus, concerns about the shortage of PPE, absence of psychological support, and the burden of long working hours (Muller et al., 2020). Additionally, one-third of our participants reported experiencing nightmares related to the virus, and this may further indicate the overwhelming affliction endured by healthcare workers during the pandemic, in consonance with similar results published by Herrero et al. (2020). A large proportion of HCPs (66.3%) also adopted a different attitude toward patients (e.g., eviction or incomplete routine clinical examination), which further highlights their fear of infection owing to the lack of PPE (45.4% of HCPs reported insufficient PPE). It is important to note that a fearful or suspicious attitude may jeopardize the quality of care provided to all types of patients.

Our findings showed that HCPs who were physically active reported fewer symptoms in all mental health measures. This result parallels findings reported by Maugeri et al. (2020), who noted a significant positive correlation between physical activity and mental wellbeing in the general population during the current pandemic. Other studies support the benefits of physical activities and show that it buffers the negative effects of bad experiences and traumatic events, and can provide resilience in facing challenges (Richards et al., 2015; Brailovskaia and Margraf, 2017). In the current pandemic, physical activity has been shown to be negatively associated with the psychological burden induced by COVID-19: those who engage in a regular exercise routine may have a less stressful pandemic experience, and thus tend to cope better with everyday distress (Brailovskaia et al., 2021).

Our results also indicated that the availability of appropriate PPE was a protective factor against depression and anxiety. This finding is consistent with a study by Zhang S.X. et al. (2020), who showed that having PPE was associated with less distress. Also, Dong and Gao (2021) identified the unavailability of PPE as a significant risk factor for both anxiety and insomnia.

In the light of our findings and as the COVID-19 pandemic continues, providing appropriate strategies will be of great importance in supporting HCPs, especially those who are involved in the treatment of patients with COVID-19. To avoid negative psychological outcomes, efforts should be tailored and directed toward high-risk groups, i.e., female nurses who have a history of psychiatric illness, and HCPs with comorbidities.

For example, female nurses working in frontline positions during the health crisis should be allowed more rest and more days off, their psychological symptoms should be regularly monitored, and the threshold for seeking psychological specialist support should be lowered. In addition, HCPs with a known psychiatric history should be screened for any aggravation of their symptoms, and care should be sought immediately if their symptoms worsen.

The use of public transportation during the pandemic was shown to be an important contributor to the mental burden borne by our participants; thus policymakers should endorse measures that favor social distancing and frequent sanitization of public transportation. If economically feasible, dedicated private transportation should be used by HCPs. Finally, to ensure that optimum healthcare is provided to patients, more advocacy tools should be implemented to persuade decision-makers and healthcare administration officials of the importance of PPE availability, which is known to be a prominent protective factor against the mental burden faced by HCPs.

Our study has several limitations. First, because this was a cross-sectional survey-based study, casual inferences are limited. Second, the possibility of selection bias was non-negligible because all HCPs were invited to participate, and those who participated may have been more aware of their mental health issues than those who did not. Third, the PHQ-2 and GAD-2 psychological scales were used mainly for screening purposes; they do not evaluate the degree or the severity of symptoms. Fourth, we did not screen for any aggravation of symptoms during the pandemic, e.g., preexisting mental health symptoms vs. new symptoms, and this limitation applies particularly to participants with a previous history of psychiatric illness. Moreover, other potential confounding variables including past or current pharmacologic treatment or data on past psychological intervention in HCPs could have impacted the outcome of the present study reflecting the mental health consequences of COVID-19 (Carvalho et al., 2016; Cosci et al., 2016; Swartz, 2020). Additionally, the 3-week duration of the data collection process may have affected the quality of the responses, given that the incidence of COVID-19 and perceptions related to infection may have differed between the first and last day of the survey.

Despite these limitations, our study provides important baseline information on many psychological morbidities endured by healthcare workers during this critical period. Our findings can serve as an important tool enabling policymakers to provide guidance to HCPs about effective strategies to protect their mental health. Because of the increasingly worrisome mental health outcomes among HCPs, their symptoms may become more severe with time, thus we recommend conducting further research on the long-term psychological implications.

## Conclusion

The COVID-19 pandemic has had a deleterious effect on healthcare workers’ mental health, with evidence of an increase in depressive, anxiety, and insomnia symptoms negatively impacting the quality of care they provide to patients. Hence, special attention should be paid to HCPs who are female nurses, and to those who have a history of psychiatric illness and are prone to mental disorders. Also, urgent measures should be implemented toward more rapid and effective risk communication, endorsement of protective factors that could help to manage this burden, and more efficient identification of those who are in distress.

## Data Availability Statement

The original contributions presented in the study are included in the article/supplementary material, further inquiries can be directed to the corresponding author.

## Ethics Statement

This study was approved by the Institutional Committee of Fattouma Bourguiba University Hospital in accordance with the Declaration of Helsinki. Electronic informed consent was obtained from the participants. The patients/participants provided their written informed consent to participate in this study.

## Author Contributions

AH and MJ conceptualized and designed the study and supervised the writing and analyses. LM and MF contributed to data collection and analysis. SO critically reviewed the manuscript. All authors approved the final manuscript as submitted.

## Conflict of Interest

The authors declare that the research was conducted in the absence of any commercial or financial relationships that could be construed as a potential conflict of interest.

## Publisher’s Note

All claims expressed in this article are solely those of the authors and do not necessarily represent those of their affiliated organizations, or those of the publisher, the editors and the reviewers. Any product that may be evaluated in this article, or claim that may be made by its manufacturer, is not guaranteed or endorsed by the publisher.

## Abbreviations

HCPs: healthcare professionals
COVID-19: novel coronavirus disease
SARS-CoV-2: severe acute respiratory syndrome coronavirus 2
PPE: personnel protective equipment
ISI: Insomnia Severity Index
PHQ-2: Two-Item Patient Health Questionnaire
GAD-2: Two-Item Generalized Anxiety Disorder instrument
MERS-CoV: Middle East respiratory syndrome coronavirus.
